# Genetic variants associated with lean and obese type 2 diabetes in a Han Chinese population: A case–control study

**DOI:** 10.1097/MD.0000000000003841

**Published:** 2016-06-10

**Authors:** Xiaomu Kong, Xiaoyan Xing, Jing Hong, Xuelian Zhang, Wenying Yang

**Affiliations:** Department of Endocrinology, China-Japan Friendship Hospital, Beijing, P.R. China.

**Keywords:** Chinese Hans, genetic variants, lean type 2 diabetes, obese type 2 diabetes

## Abstract

Type 2 diabetes (T2D) is highly phenotypically heterogeneous. Genetics of the heterogeneity of lean and obese T2D is not clear. The aim of the present study was to identify the associations of T2D-related genetic variants with the risks for lean and obese T2D among the Chinese Han population. A case–control study consisting of 5338 T2D patients and 4663 normal glycemic controls of Chinese Han recruited in the Chinese National Diabetes and Metabolic Disorders Study was conducted. T2D cases were identified according to the 1999 World Health Organization criteria. Lean T2D was defined as T2D patient with a body mass index (BMI) <23 kg/m^2^, whereas obese T2D was defined as T2D patient with a BMI ≥28 kg/m^2^. Twenty-five genome-wide association studies previously validated T2D-related single-nucleotide polymorphisms (SNPs) were genotyped. A genotype risk score (GRS) based on the 25 SNPs was created. After adjusting for multiple covariates, SNPs in or near *CDKAL1*, *CDKN2BAS*, *KCNQ1*, *TCF7L2*, *CDC123/CAMK1D*, *HHEX*, and *TCF2* were associated with the risk for lean T2D, and SNPs in or near *KCNQ1* and *FTO* were associated with the risk for obese T2D. The results showed that the GRS for 25 T2D-related SNPs was more strongly associated with the risk for lean T2D (*P*_trend_ = 2.66 × 10^−12^) than for obese T2D (*P*_trend_ = 2.91 × 10^−5^) in our study population. Notably, the T2D GRS contributed to lower obesity-related measurements and greater β-cell dysfunction, including lower insulin levels in oral glucose tolerance test, decreased insulinogenic index, and Homeostasis Model Assessment for β-cell Function. In conclusion, our findings identified T2D-related genetic loci that contribute to the risk of lean and obese T2D individually and additively in a Chinese Han population. Moreover, the study highlights the contribution of known T2D genomic loci to the heterogeneity of lean and obese T2D in Chinese Hans.

## Abstract 2

Supplemental Digital Content is available in the text

## 1 Introduction

The prevalence of diabetes has increased dramatically worldwide in recent decades.^[1]^ The Chinese National Diabetes and Metabolic Disorders Study (DMS) conducted during 2007 to 2008 reported that the prevalence of diabetes in China was 9.7% among adults, and more than 90% of the affected individuals had type 2 diabetes (T2D).^[2]^ A more recent study indicated that the prevalence of diabetes among Chinese adults had increased to 11.6% by 2010.^[3]^

As a common disease, T2D is highly phenotypically heterogeneous.^[1,4]^ The most common feature of classical T2D patients is obesity, but the prevalence of the disease in underweight and normal-weight lean patients has received more attention in recent years.^[4]^ Studies have indicated that lean patients with T2D exhibit more rapid, early loss of β-cell function while still having low levels of insulin resistance in contrast to obese patients with T2D, and thus, many require early insulin treatment.^[1,4]^ Notably, in East Asian populations, T2D onset occurs in patients with a lower mean body mass index (BMI) compared with that of Caucasian patients, and T2D diabetes among East Asian populations is characterized by early β-cell dysfunction in the setting of insulin resistance, which suggests that the prevalence of lean T2D is higher among East Asians, including the Chinese Han population.^[1,5,6]^ Therefore, the identification and comparison of risk factors for lean and obese T2D is of significant importance for the prediction and management of T2D in Chinese Han patients.

The complicated pathogenesis of T2D in lean and obese patients involves both genetic and environmental factors.^[7]^ Genome-wide association studies (GWAS) employing high-throughput technologies and primarily involving Caucasian patients have revealed that more than 120 genomic loci are associated with T2D, and similar findings have been replicated in different populations.^[8]^ In our previous study, we confirmed the association of single-nucleotide polymorphisms (SNPs) in or near *WFS1*, *CDKAL1*, *CDKN2A/2B*, *CDC123/CAMK1D*, *HHEX*, *TCF7L2*, *KCNQ1*, and *MTNR1B* with T2D in the Chinese population evaluated in the DMS.^[9]^ However, our understanding of the genetics of clinically heterogeneous T2D has not greatly improved.^[4]^ Previous studies identified potential risk variants that may predispose patients to lean T2D versus obese T2D.^[10–14]^ Moreover, a recent GWAS in Caucasians identified 2 novel genomic loci (*LAMA1*, *HMG20A*) that were associated with the risk for lean T2D (BMI < 25 kg/m^2^) and obese T2D (BMI ≥30 kg/m^2^).^[12]^ The heterogeneity of T2D within patient groups stratified by BMI was less than that among all cases, which possibly increased the statistical power of the genetic study.^[12]^ Previous studies also have suggested that insulin secretory variants confer a greater risk for T2D in lean individuals, whereas insulin sensitivity variants more significantly modulate the T2D risk in obese subjects.^[10–14]^ For example, the *TCF7L2* genetic variant was found to be more strongly associated with T2D in lean individuals, whereas the *FTO* genetic variant was more strongly associated with T2D in obese individuals.^[10,12]^

Considering the large proportion of lean individuals among Chinese T2D patients, a clearer understanding of the genetics of lean versus obese T2D in Chinese Han patients can support better management of risk factors in these patients. A previous study conducted in the Chongqing city of China confirmed the associations of 6 genetic loci with the risk for T2D in lean individuals (BMI 22.96 ± 1.64 kg/m^2^).^[15]^ We speculated that the genes responsible for susceptibility to T2D may differ between lean and obese Chinese Han patients and that the known T2D genomic loci, most of which are related to β-cell function, may contribute primarily to the genetic predisposition for T2D among lean Chinese Han individuals.

In the present study, we examined the associations of 25 SNPs in established T2D-related genomic loci individually and additively with the risks for lean and obese T2D among Chinese individuals included in the DMS, and also with obesity-related and glycemic quantitative traits in T2D patients. The findings of the present study highlight the contributions of known T2D genomic loci to the heterogeneity of T2D in lean and obese Chinese Han patients.

## 2 Methods

### 2.1 Study participants and definitions of lean and obese T2D

All study participants were enrolled in the DMS.^[2]^ The study protocol was approved by the Ethics Committee of the China–Japan Friendship Hospital in Beijing. The study was performed in accordance with the Declaration of Helsinki II. Written informed consent was obtained before data collection.

Type 2 diabetes was defined by World Health Organization (WHO) 1999 criteria of a fasting plasma glucose (FPG) ≥7.0 mmol/L and/or a 2-hour oral glucose tolerance test (OGTT) plasma glucose ≥11.1 mmol/L, or a self-reported history of T2D. Therefore, 5338 T2D patients and 4663 controls were included in the analysis.^[9]^

Lean T2D was defined as T2D in patients with a BMI <23 kg/m^2^,^[16]^ whereas obese T2D was defined as T2D in patients with a BMI ≥28 kg/m^2^.^[17]^ Of the T2D patients included in the present analysis, 1125 were lean and 1399 were obese.

For the normal glycemic controls, we only included the participants aged over 40, who were with normal glycemic regulation (FPG < 6.1 mmol/L and 2-hour OGTT plasma glucose < 7.8 mmol/L), and no family history and personal history of diabetes, BMI <28 kg/m^2^, blood pressure below 140/90, and normal blood triglyceride (<1.7 mmol/L) and high-density lipoprotein-cholesterol (≥1.0 mmol/L) levels.

### 2.2 Clinical measurements and laboratory methods

Body weight, height, waist circumference (WC), and hip circumference (HC) were measured using standard methods. BMI was calculated as weight/height^2^ (kg/m^2^). The waist-hip-ratio (WHR) was calculated. Each participant completed a standard 75-g OGTT after overnight fasting. Plasma glucose and serum insulin levels at 0, 30, and 120 minutes during the OGTT were tested as previous described.^[18]^ β-cell function was estimated using the Homeostasis Model Assessment for β-cell Function (HOMA-B) and insulinogenic indices, and insulin resistance was assessed by the Homeostasis Model Assessment for Insulin Resistance (HOMA-IR) and Matsuda index (ISIm). The indices were calculated as previously described using the following formulae:

HOMA-B = fasting serum insulin (mU/L) × 20 /(FPG [mmol/L] − 3.5)^[19]^

Insulinogenic index = (30-minute OGTT insulin [mU/L]  − fasting serum insulin [mU/L])/(30-minute OGTT glucose [mmol/L] − FPG [mmol/L])^[20]^

HOMA-IR = fasting serum insulin (mU/L) × FPG (mmol/L)/22.5^[19]^

ISIm = 10,000/(FPG [mg/dL] × fasting serum insulin [mU/L] × mean OGTT glucose [mg/dL] × mean OGTT insulin [mU/L])^1/2^^[21]^

### 2.3 Genotyping

Genomic DNA was directly isolated from human peripheral blood samples. Thirty-one T2D-related SNPs validated by previous GWAS were selected and genotyped in the participants using the Illumina GoldenGate Indexing assay (Illumina Inc., San Diego, CA)^[13,22–33]^. Before further analysis, we excluded SNPs including rs13266634, rs231362, rs5945326, and rs1531343, because their genotyping success rates were lower than 90%. rs7957197 and rs7578597, of which the minor allele frequency (MAF) was less than 0.01, were also excluded. Finally, it achieved a 98.55% overall mean call rate of the remaining 25 SNPs, and also a high concordance rate (100%) based on 229 genotyping duplication. Supplemental Table 1 shows the detailed information of each genotyped SNP.

### 2.4 Statistical analysis

Chi-square test was used to examine the Hardy–Weinberg equilibrium for each SNP in the present population (Supplemental Table 1). Logistic regression analysis was used to test associations of SNPs with the risk for lean or obese T2D assuming an additive genetic model, which was also applied in other analyses as appropriate. Before further analysis, non-Gaussian distributed quantitative traits were natural logarithmically transformed to normal distributions. The associations between SNPs and quantitative traits were examined using linear regression model. For each comparison, 2 multivariable models were applied: model 1, age and sex were adjusted as covariables; and model 2, age, sex, and BMI were adjusted. In each individual without missing genotyping data (T2D: n = 4371; control: n = 4032), genotype risk scores (GRS) of SNPs were constructed using the sum of the reported risk alleles for T2D. The risks for lean and obese T2D, and also the quantitative traits, were compared among GRS quartiles in T2D patients. The associations of GRS with the risks for lean and obese T2D were further tested in the logistic model which included sex, age, BMI, and the identified risk factors for T2D in Chinese (including education, waist circumference, resting heart rate, SBP, triglyceride, and residence^[2]^), and the validity of the models were provided (Supplemental Table 2). Moreover, to eliminate the potential influence of hypoglycemic treatments, we then conducted the sensitivity analyses by only including the newly diagnosed T2D patients (n = 2731) and controls (n = 4032) from the present population. For single SNP analyses, Bonferroni correction was used to correct multiple comparisons, and *P* values less than 0.002 (0.05/25) were considered statistically significant. In addition, *P* values between 0.002 and 0.05 were defined as nominal significant, and *P* values between 0.05 and 0.10 were defined as marginal significant.^[34,35]^*P* values less than 0.05 were considered statistically significant for T2D GRS analyses. Statistical analyses were performed using SAS (version 9.3; SAS Institute, Cary, NC) and PLINK software (v1.05; http://pngu.mgh.harvard.edu/purcell/plink).^[36]^

## 3 Results

### 3.1 Clinical demographics of the study population

The clinic characteristics of the DMS population are presented in Table 1. Compared with the normal glycemic control group, the T2D group included more male patients and patients of older age. As expected, the prevalence of metabolic disorders related to glucose, blood pressure, and lipids, and also obesity was higher among T2D patients.

**Table 1:**
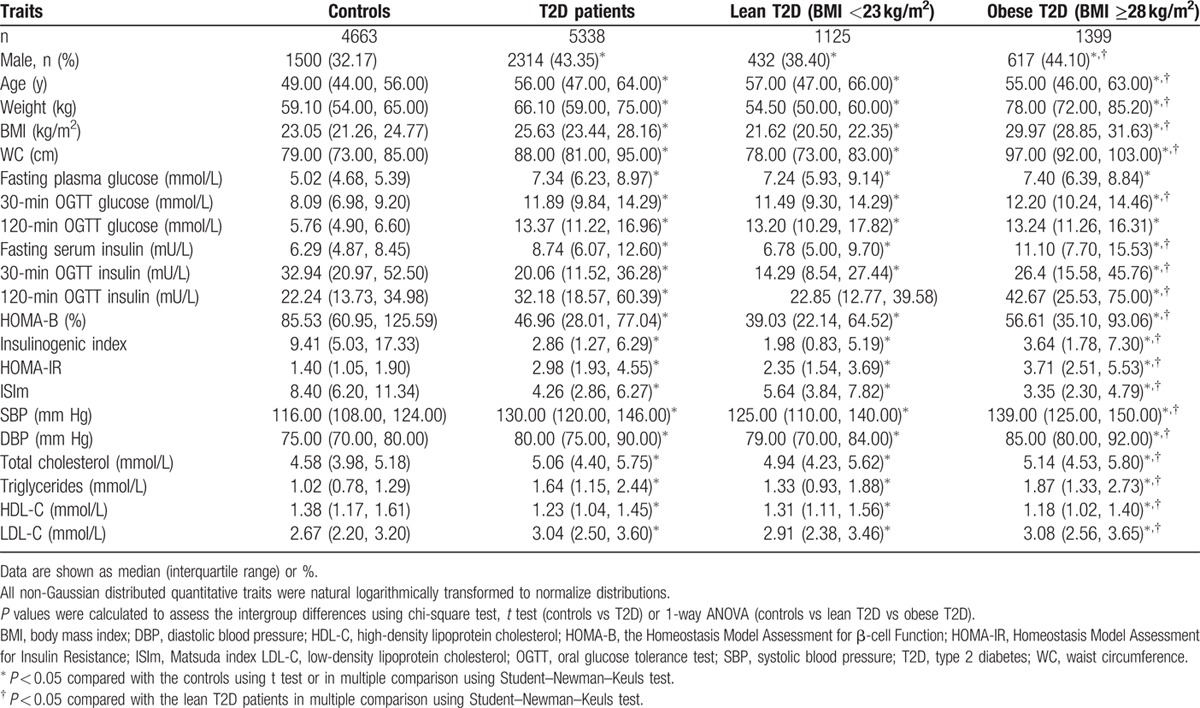
Clinical characteristics of the study population.

Compared with the obese T2D group (BMI ≥28 kg/m^2^), the lean T2D group (BMI <23 kg/m^2^) included more female patients, and also older patients. Notably, although the fasting glucose and 2-hour glucose during OGTT results were comparable between the 2 groups, the obese T2D group showed greater fasting and glucose-induced insulin secretion during OGTT, whereas greater β-cell dysfunction and better insulin sensitivity were observed in the lean T2D group. In addition, the obese T2D patients exhibited higher blood pressure and more severe lipid disorder.

### 3.2 T2D-related SNPs associated with the risks for lean and obese T2D in Chinese Han individuals

As shown in Table 2, after adjustment for covariates, rs7756992 in *CDKAL1*, rs10811661 near *CDKN2BAS*, and rs2237895 in *KCNQ1* were significantly associated with the risk for lean T2D (odds ratios [ORs] 1.20–1.28, *P* values 5.51 × 10^−6^ to 2.88 × 10^−4^), and these remained significant after Bonferroni correction for multiple comparisons (*P* < 2.00 × 10^−3^). Rs7903146 in *TCF7L2*, rs12779790 near *CDC123/CAMK1D*, rs1111875 near *HHEX*, and rs7501939 in *TCF2* showed nominal associations with the risk for lean T2D (ORs 1.12–1.28, *P* values 1.52 × 10^−2^ to 3.34 × 10^−2^). Given further adjustment for BMI, the associations of rs7756992, rs10811661, rs2237895, and rs7501939 with the risk for lean T2D were unaffected (*P* values 2.60 × 10^−6^ to 1.96 × 10^−2^). In addition, rs7903146, rs12779790, and rs1111875 showed marginal associations with the risk for lean T2D after adjustment for BMI (*P* values 5.92 × 10^−2^ to 7.51 × 10^−2^).

**Table 2:**
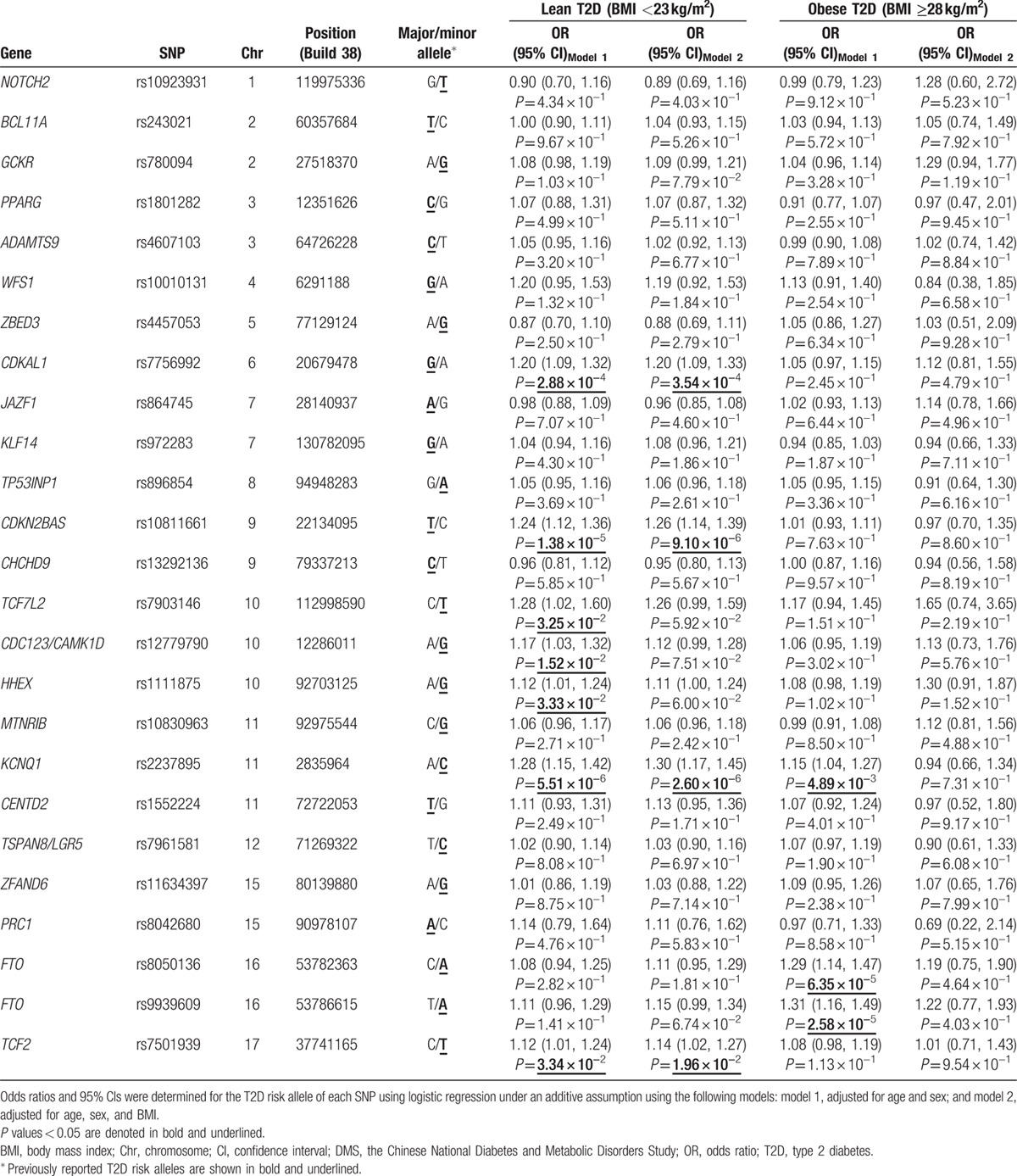
Associations of type 2 diabetes-related genetic variants with the risks for lean and obese type 2 diabetes among patients of Chinese ancestry.

Rs2237895 in *KCNQ1* and rs8050136 and rs9939609 in *FTO* were associated with the risk for obese T2D (ORs 1.15–1.31, *P* values 2.58 × 10^−5^ to 4.89 × 10^−3^). The associations between SNPs in *FTO* and the risk for obese T2D remained significant after Bonferroni correction (*P* < 2.00 × 10^−3^), but after adjustment for BMI, the associations were attenuated (Table 2).

Among the SNPs listed above, only rs2237895 in *KCNQ1* was associated with the risks for both lean T2D and obese T2D, and the corresponding OR was greater for lean T2D (OR 1.28) than for obese T2D (OR 1.15).

### 3.3 Associations of T2D GRS with the risks for lean and obese T2D in chinese Han individuals

Joint effect analysis showed that the GRSs of 25 T2D-related SNPs were significantly associated with the risk for lean T2D (*P*_trend_ = 2.66 × 10^−12^), and also the risk for obese T2D (*P*_trend_ = 2.91 × 10^−5^; Table 3). Compared with that for the lowest quartile of GRS, the ORs (95% confidence intervals [CIs]) for the risk of lean T2D were 1.14 (0.88–1.47, *P* = 3.12 × 10^−1^); 1.33 (1.08–1.63, *P* = 6.60 × 10^−3^); and 1.82 (1.50–2.21, *P* = 1.41 × 10^−9^) for the other 3 quartiles, and these were not significantly altered upon adjustment for BMI. No significant associations were observed between the T2D GRS quartiles and the risk for obese T2D except for the highest quartile which showed an OR (95% CI) of 1.32 (1.12–1.57, *P* = 1.34 × 10^−3^). However, the associations of the T2D GRS and the highest GRS quartile with the risk for obese T2D were attenuated to nonsignificant after adjustment for BMI. Moreover, for the setting quartiles, the ORs for lean T2D were much higher than those for obese T2D.

**Table 3:**
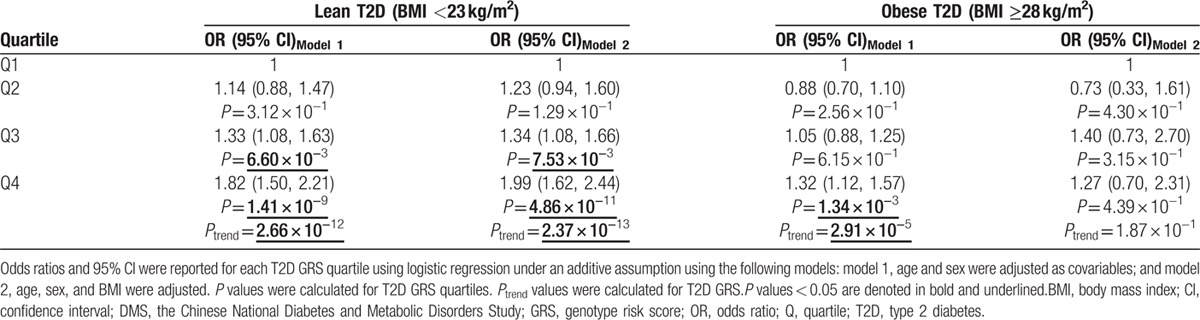
Associations of type 2 diabetes genotype risk score with the risks for lean and obese type 2 diabetes among patients of Chinese ancestry.

When we examined the associations of T2D GRS with the risk for lean and obese T2D in the newly diagnosed T2D patients to eliminate the effect of glucose-lowering treatment, the above findings were further confirmed (Supplemental Table 3).

### 3.4 Associations of T2D GRS with the quantitative traits in Chinese Han patients with T2D

A higher T2D GRS was found to significantly contribute to a lower body weight (β [SE] −0.0031 [0.0008], *P* = 2.01 × 10^−4^), BMI (−0.0024 [0.0008], *P* = 1.80 × 10^−3^), WC (−0.0017 [0.0006], *P* = 4.28 × 10^−3^), and WHR (−0.0007 [0.0004], *P* = 5.75 × 10^−2^) in Chinese Han T2D patients, but these associations attenuated to nonsignificant after adjustment for BMI (Table 4).

**Table 4:**
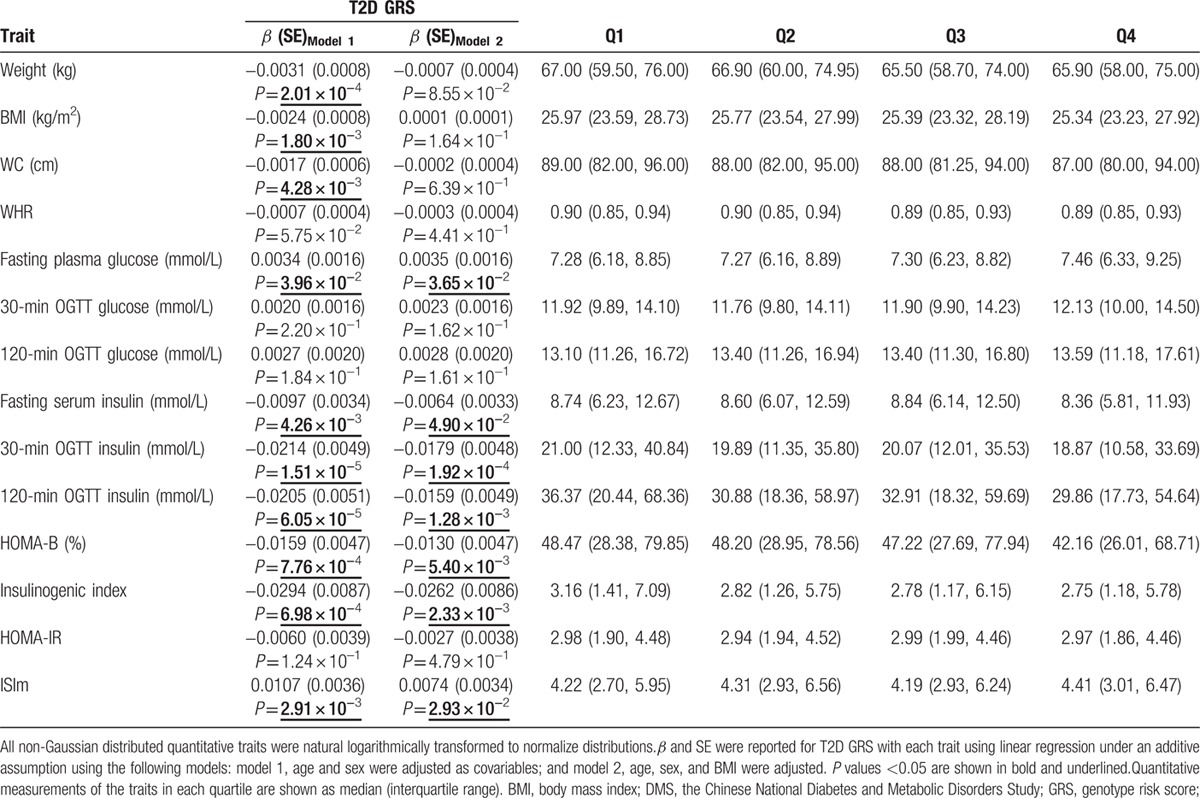
Associations of type 2 diabetes genotype risk score with the obesity-related and glycemic quantitative traits in type 2 diabetes patients of Chinese ancestry.

Beyond the obesity-related traits, T2D individuals with a higher GRS showed a higher fasting blood glucose (0.0034 [0.0016], *P* = 3.96 × 10^−2^). Moreover, the GRS were related to the lower fasting insulin level (−0.0097 [0.0034], *P* = 4.26 × 10^−3^) and postprandial insulin level (30-minute insulin: −0.0214 [0.0049], *P* = 1.51 × 10^−5^; 2-hour insulin: −0.0205 [0.0051], *P* = 6.05 × 10^−5^). Notably, T2D patients with a higher T2D GRS showed greater β-cell dysfunction (HOMA-B: −0.0159 [0.0047], *P* = 7.76 × 10^−4^; insulinogenic index: −0.0294 [0.0087], *P* = 6.98 × 10^−4^) and better insulin sensitivity (0.0107 [0.0036], *P* = 2.91 × 10^−3^). Furthermore, these findings were not significantly altered by BMI adjustment (Table 4).

The associations of T2D GRS with the quantitative traits in the newly diagnosed T2D patients were tested in sensitivity analysis to eliminate the effect of glucose-lowering treatment. Similar results were achieved and shown in Supplemental Table 4.

To clarify the contribution of individual SNPs to quantitative traits in T2D patients, we also performed an association study between each SNP and trait. The associations identified are listed in Supplemental Table 5. The T2D risk alleles of genetic variants in *BCL11A*, *PPARG*, *CDKAL1*, *CDKN2BAS*, *CHCHD9*, *CDC123/CAMK1D*, *MTNR1B*, and *KCNQ1* were related to a decreased measurement of at least one of the obesity-related traits (body weight, BMI, WC, WHR), whereas the T2D risk alleles of genetic variants of *WFS1*, *TP53INP1*, and *FTO* were associated with a higher measurement. The T2D risk alleles of genetic variants from *GCKR*, *KCNQ1*, and *CENTD2* were related to the higher fasting or postprandial glucose levels. The T2D risk alleles of genetic variants in *PPARG*, *WFS1*, *CDKAL1*, *CDKN2BAS*, *CENTD2*, *ZFAND6*, and *TCF2* were associated with lower fasting or postprandial insulin secretion. The T2D risk alleles of genetic variants in *BCL11A*, *PPARG*, *WFS1*, *CDKN2BAS*, *HHEX*, *KCNQ1*, and *CENTD2* were related to greater β-cell dysfunction (HOMA-B, insulinogenic index), and the T2D risk allele of the genetic variant in *PPARG* was related to better insulin sensitivity (ISIm).

## 4 Discussion

By comparing patients with either lean or obese BMI to the full set of normal glycemic controls in the DMS, the present study identified that genetic variants in or near *CDKAL1*, *CDKN2BAS*, *KCNQ1*, *TCF7L2*, *CDC123/CAMK1D*, *HHEX*, and *TCF2* were associated with the risk for lean T2D, and genetic variants in or near *KCNQ1* and *FTO* were associated with the risk for obese T2D in Chinese Han patients. Through the T2D GRS of the 25 T2D genetic loci, we further discovered that lean T2D patients have a stronger genetic predisposition for T2D risk alleles than did obese T2D patients among the Chinese Han population. Moreover, the T2D GRS contributed to the lower obesity-related measurements and greater β-cell dysfunction in T2D patients. To the best of our knowledge, this is the first study to investigate associations between known T2D genomic loci and the risk for lean and obese T2D in a Chinese Han population.

Type 2 diabetes is a common disease with high heterogeneity.^[1,4]^ Epidemiological studies have demonstrated that underweight or normal-weight T2D (lean T2D) patients tend to develop rapid β-cell failure in the condition of insulin sensitivity and require early insulin treatment.^[1,4]^ Several studies examined the genetic heterogeneity in lean and obese T2D patients in Caucasian populations previously.^[10–14,37]^ These studies indicated that the lean T2D patients were enriched for known T2D risk alleles in comparison with obese T2D patients,^[10–14]^ most of these were related to β-cell function. It was also observed that most of the insulin secretion-related variants, including SNPs from *TCF7L2*, *CDKN2BAS*, *CDKAL1*, *HHEX*, and so on, showed a larger effective size for the risk of lean T2D than for the risk of obese T2D, and the insulin sensitivity-related variants (*PPARG*, *FTO*, etc) showed larger effective sizes for the risk of obese T2D.^[10–14]^ Recently, 2 novel genomic loci, *LAMA1* and *HMG20A*, were identified by 2 independent GWAS in lean (BMI <25 kg/m^2^) and obese individuals (BMI ≥30 kg/m^2^) of Caucasian populations, respectively, partly due to the strategy by which cases were included, which reduced the heterogeneity of T2D, resulting in an increased statistical power.^[12]^ In addition, 6 genomic loci associated with fasting insulin and glucose were discovered in a Caucasian population via a novel joint meta-analytical approach that accounted for BMI and the potential interaction between BMI and genetic variants.^[37]^ Although a previous study did include the lean Chinese Han population,^[15]^ the genetics of lean and obese T2D in Chinese Han patients were still not clear.

In the current study, we defined lean and obese T2D using BMI cut-off values determined to be optimal for the Chinese Han population.^[16,17]^ Our study identified SNPs in or near *CDKAL1*, *CDKN2BAS*, and *KCNQ1* as significantly related to the risk for lean T2D with effective sizes ranging from 1.20 to 1.28, values which were obviously higher than the effective size for obese T2D (ORs 1.01–1.15). Previously, both in vitro and rodent studies demonstrated that the gene products of *CDKAL1*, *CDKN2BAS*, and *KCNQ1* are expressed in pancreatic β-cells and have critical roles in β-cell survival and function.^[38–42]^ Moreover, SNPs in or near *TCF7L2*, *CDC123/CAMK1D*, *HHEX*, and *TCF2* showed robust associations with the risk for lean T2D, but not with the risk for obese T2D. In contrast, SNPs near *KCNQ1* and in *FTO* were associated with obese T2D and mediated via obesity. The effective sizes for *FTO* on the risk for obese T2D (OR 1.29–1.31) were higher than those for the lean T2D (OR 1.08–1.11). *FTO* is expressed in the hypothalamus and modulates food intake and obesity, which is closely linked to insulin resistance.^[43]^ Therefore, the results of our present study confirm that the genomic loci related to secretion tend to predispose lean Chinese Han individuals to T2D, whereas the insulin sensitivity-related genomic loci showed stronger association with obese T2D.

Our GRS study of the 25 T2D SNPs further confirmed the additive effects of the T2D SNPs. The T2D GRS showed a much greater effective size on the risk for lean T2D than for obese T2D in our Chinese Han population, and this association was not affected by adjustment according to BMI. Moreover, as expected, T2D patients with a higher T2D GRS were leaner and had worse β-cell function. These results suggest that lean T2D patients carry a higher dose of T2D risk alleles, which leads to worse β-cell function at the same time. Our findings also show that β-cell dysfunction has a critical role in the pathogenesis of T2D in Chinese individuals.

Interestingly, a previous study showed that the genetic variants that predispose individuals to obesity also contributed to an increased risk for T2D in Caucasians, and this conclusion was reached by calculating an obesity GRS for 12 GWAS-validated BMI-related risk alleles,^[44]^ which provided insight into the genetics of obese T2D. We previously demonstrated that the obesity risk alleles of genetic variants from *MC4R* and *GNPDA2* also contribute to an increased risk for T2D in Chinese individuals.^[18]^ However, whether such obesity-related genomic loci are the major genetic factors for obese T2D in the Chinese Han population is still under investigation.

Notably, ethnic discrepancies in the clinical features of T2D between East Asians (including Chinese Hans) and Caucasians have been established.^[1]^ East Asians develop T2D at a lower BMI and show earlier β-cell dysfunction compared with Caucasians. Our previous study in a Chinese Han population confirmed the associations of T2D with GWAS-validated SNPs in or near *WFS1*, *CDKAL1*, *CDKN2A/2B*, *CDC123/CAMK1D*, *HHEX*, *TCF7L2*, *KCNQ1*, and *MTNR1B*, all of which were essential in β-cell function.^[9]^ However, many genetic variants have been revealed by GWAS in Caucasians that could not be replicated in Chinese Han or other East Asian populations, partly due to the heterogeneity of T2D. Because the proportion of T2D patients who are lean is relatively higher among East Asians compared with Caucasians, the genetic variants that predispose individuals to lean T2D may be easier to be detected in East Asian populations. A previous study suggested that BMI-based stratification of T2D cases can increase the statistic power to replicate strong T2D associations that have been masked by the heterogeneity of T2D at the same sample size.^[12]^ Therefore, by stratifying T2D patients according to BMI, the current study successfully identified susceptibility genes for lean and obese T2D in a Chinese Han population. In the future, a GWAS using BMI stratification will be useful for identifying novel genomic loci for T2D in the Chinese population.

The present study has the following strengths. Most importantly, this is the first study to investigate the genetics of lean and obese T2D in Chinese Han patients and to partly explain the genetic heterogeneity of T2D. Second, the study population from the DMS is considered representative of the national population, as it is ethnically homogeneous and includes a relatively large population of Chinese Hans, and thus, the results can be well generalized to China mainland. Third, the optimal cut-off values for BMI for Chinese individuals were used to define lean and obese T2D. Finally, ethnic discrepancies related to T2D were considered along with their genetic basis. Our findings suggest that the β-cell function-related genetic factors are major contributors to the T2D risk of East Asians who are leaner and exhibit poor β-cell function.

However, our study also has several limitations. First, it is a replicative study of known T2D loci, and thus, the results do not consider uncovered T2D risk alleles across the genome. However, the GWAS-validated genomic loci could be the gene region showing the strongest association with T2D, and the strategy of stratifying T2D cases according to BMI can be applied to identify novel susceptibility genes based on the risk for heterogeneous T2D in the future. Second, some suspect that genetic variants have a stronger effective size in Asians compared with Caucasians.^[45]^ However, in the present study, we were unable to compare the many risk alleles and their effective sizes between ethnicities. Thus, future genetic studies are warranted to further clarify the ethnic discrepancies. In addition, there could be a potential contamination of type 1 diabetes (e.g., latent autoimmune diabetes in adults) in the participants. However, its proportion was quite low among the diabetes population of Chinese aged over 20 years; thus it was unlikely to affect the results.

In conclusion, we identified significant associations of genetic variants in or near *CDKAL1*, *CDKN2BAS*, and *KCNQ1* with the risk for lean T2D among Chinese Han individuals, and also the associations of genetic variants near *KCNQ1* or in *FTO* with the risk for obese T2D. T2D-related risk alleles showed a stronger predisposition to lean T2D than to obese T2D in Chinese Hans. Accordingly, T2D patients with a higher T2D GRS were leaner or had worse β-cell function. The present study improves our understanding of the heterogeneity of T2D in the Chinese Han population and highlights the importance of genetic heterogeneity in elucidating the pathogenesis mechanisms of T2D, which could provide an explanation for ethnic discrepancies.

## Supplementary Material

Supplemental Digital Content
