# Supplementary material for: Genetic variants associated with lean and obese type 2 diabetes in a Han Chinese population: A case–control study
Source: Medicine (Baltimore). 2016 Jun 10;95(23):e3841. doi: 10.1097/MD.0000000000003841 (PMC4907669; doi:10.1097/MD.0000000000003841)
Supplement: Supplemental Digital Content [file medi-95-e3841-s001.doc]

**Supplemental Table 1. Information for the genotyped genetic variants in the present study.**

| **SNP** | **Chr.** | **Position** | **Major/minor allele^a^** | **Gene** | **Localization** | **HW-*P_Control_*** | **HW-*P_T2D_*** | **MAF** | | | |
| --- | --- | --- | --- | --- | --- | --- | --- | --- | --- | --- | --- |
|  |  | **(Build 38)** |  |  |  |  |  | **Control^b^** | **T2D^b^** | **CEU^c^** | **CHB^c^** |
| rs10923931 | 1 | 119975336 | G/**T** | *NOTCH2* | Intronic variant | 0.848 | 0.238 | 0.040 | 0.036 | 0.058 | 0.042 |
| rs243021 | 2 | 60357684 | **T**/C | *BCL11A^d^* | Intergenic variant | 0.735 | 0.631 | 0.320 | 0.312 | 0.450 | 0.375 |
| rs780094 | 2 | 27518370 | A/**G** | *GCKR* | Intronic variant | 0.359 | 0.847 | 0.476 | 0.490 | 0.400 | 0.400 |
| rs1801282 | 3 | 12351626 | **C**/G | *PPARG* | Coding, non-synonymous | 1.000 | 1.000 | 0.065 | 0.061 | 0.092 | 0.050 |
| rs4607103 | 3 | 64726228 | **C**/T | *ADAMTS9^d^* | Intergenic variant | 0.052 | 0.361 | 0.377 | 0.370 | 0.200 | 0.425 |
| rs10010131 | 4 | 6291188 | **G**/A | *WFS1* | Intronic variant | 0.406 | 0.026 | 0.046 | 0.039 | 0.333 | 0.017 |
| rs4457053 | 5 | 77129124 | A/**G** | *ZBED3^d^* | Intergenic variant | 0.535 | 0.295 | 0.051 | 0.049 | 0.317 | 0.025 |
| rs7756992 | 6 | 20679478 | **G**/A | *CDKAL1* | Intronic variant | 0.044 | 0.578 | 0.477 | 0.452 | 0.250 | 0.483 |
| rs864745 | 7 | 28140937 | **A**/G | *JAZF1* | Intronic variant | 1.000 | 0.675 | 0.239 | 0.237 | 0.500 | 0.242 |
| rs972283 | 7 | 130782095 | **G**/A | *KLF14^d^* | Intergenic variant | 0.187 | 0.784 | 0.279 | 0.279 | 0.458 | 0.333 |
| rs896854 | 8 | 94948283 | G/**A** | *TP53INP1* | Intronic variant | 0.017 | 0.029 | 0.341 | 0.348 | 0.492 | 0.267 |
| rs10811661 | 9 | 22134095 | **T**/C | *CDKN2BAS^d^* | Intergenic variant | 0.535 | 0.230 | 0.476 | 0.440 | 0.258 | 0.442 |
| rs13292136 | 9 | 79337213 | **C**/T | *CHCHD9^d^* | Intergenic variant | 0.603 | 0.813 | 0.094 | 0.097 | 0.058 | 0.083 |
| rs7903146 | 10 | 112998590 | C/**T** | *TCF7L2* | Intronic variant | 0.324 | 0.202 | 0.039 | 0.051 | 0.308 | 0.029^e^ |
| rs12779790 | 10 | 12286011 | A/**G** | *CDC123/CAMK1D^d^* | Intergenic variant | 0.748 | 0.302 | 0.165 | 0.179 | 0.225 | 0.133 |
| rs1111875 | 10 | 92703125 | A/**G** | *HHEX^d^* | Intergenic variant | 1.000 | 0.055 | 0.284 | 0.305 | 0.408 | 0.342 |
| rs10830963 | 11 | 92975544 | C/**G** | *MTNRIB* | Intronic variant | 0.343 | 0.500 | 0.413 | 0.429 | 0.217 | 0.450 |
| rs2237895 | 11 | 2835964 | A/**C** | *KCNQ1* | Intronic variant | 0.351 | 0.001 | 0.320 | 0.357 | 0.358 | 0.300 |
| rs1552224 | 11 | 72722053 | **T**/G | *CENTD2* | Intronic variant | 0.325 | 0.472 | 0.090 | 0.083 | 0.133 | 0.092 |
| rs7961581 | 12 | 71269322 | T/**C** | *TSPAN8/LGR5* | Intronic variant | 0.148 | 0.154 | 0.205 | 0.215 | 0.250 | 0.175 |
| rs11634397 | 15 | 80139880 | A/**G** | *ZFAND6^d^* | Intergenic variant | 0.363 | 0.113 | 0.099 | 0.101 | 0.383 | 0.058 |
| rs8042680 | 15 | 90978107 | **A**/C | *PRC1* | Intronic variant | 1.000 | 0.088 | 0.019 | 0.018 | 0.242 | 0.000 |
| rs8050136 | 16 | 53782363 | C/**A** | *FTO* | Intronic variant | 0.271 | 0.428 | 0.113 | 0.129 | 0.450 | 0.150 |
| rs9939609 | 16 | 53786615 | T/**A** | *FTO* | Intronic variant | 0.417 | 0.393 | 0.113 | 0.130 | 0.450 | 0.150 |
| rs7501939 | 17 | 37741165 | C/**T** | *TCF2* | Intronic variant | 0.791 | 0.499 | 0.264 | 0.283 | 0.425 | 0.292 |

CEU = European, CHB = Han Chinese, Chr = chromosome, HW-*P_Control_* = Hardy-Weinberg equilibrium in controls, HW-*P_T2D_* = Hardy-Weinberg equilibrium in type 2 diabetes, MAF = minor allele frequency, SNP = single nucleotide polymorphism, T2D = type 2 diabetes.

^a^ Previously reported risk alleles for T2D are shown in bold.

^b^ The allele frequencies of the minor allele in the present study.

^c^ Allele frequencies of the minor allele in CHB and CEU populations of 1000 Genome Project.

^d^ The nearest gene is provided if a SNP is located in the intergenic region.

Supplemental Table 2. Associations of type 2 diabetes genotype risk score with the risks for lean and obese type 2 diabetes among patients of Chinese ancestry adjusted for gender, age, education, BMI, waist circumference, resting heart rate, SBP, triglyceride, and residence.

| **Lean T2D (BMI < 23 kg/m^2^)** | | | | | **Obese T2D (BMI ≥ 28 kg/m^2^)** | | | | | |
| --- | --- | --- | --- | --- | --- | --- | --- | --- | --- | --- |
| **OR(95%CI)** | **R-square** | **C statistic** | **Hosmer-Lemeshow test** | | **OR(95%CI)** | **R-square** | **C statistic** | **Hosmer-Lemeshow test** | |  |
|  |  |  | **Chi-square** | ***P*** |  |  |  | **Chi-square** | ***P*** |  |
| 1.11 (1.05,1.16) | 0.317 | 0.901 | 12.327 | 0.137 | 1.11 (0.41,2.99) | 0.580 | 1.000 | 0.004 | 1.000 |  |
| *P_trend_* = **1.53×10^-4^** |  |  |  |  | *P_trend_* = 8.34×10^-1^ |  |  |  |  |  |

BMI = body mass index, CI = confidence interval, DMS = the Chinese National Diabetes and Metabolic Disorders Study, GRS = genotype risk score, OR = odds ratio, SBP = systolic blood pressure, T2D = type 2 diabetes.

OR and 95% CI were reported for T2D GRS using logistic regression under an additive assumption adjusted for gender, age, education, BMI, waist circumference, resting heart rate, SBP, triglyceride, and residence. *P_trend_* values were calculated for T2D GRS.

*P* values < 0.05 are denoted in bold and underlined.

Supplemental Table 3. Associations of type 2 diabetes genotype risk score with the risks for lean and obese type 2 diabetes among the hypoglycemic treatment naive patients of Chinese ancestry.

| **Quartile** | **Lean T2D (BMI < 23 kg/m^2^)** | | **Obese T2D (BMI ≥ 28 kg/m^2^)** | |
| --- | --- | --- | --- | --- |
|  | **OR (95%CI)_Model 1_** | **OR (95%CI)_Model 2_** | **OR (95%CI)_Model 1_** | **OR (95%CI)_Model 2_** |
| **Q1** | 1 | 1 | 1 | 1 |
| **Q2** | 1.12 (0.83,1.53) | 1.21 (0.88,1.67) | 0.81 (0.62,1.05) | 0.74 (0.33,1.66) |
|  | *P* = 4.56×10^-1^ | *P* = 2.43×10^-1^ | *P* = 1.12×10^-1^ | *P* = 4.67×10^-1^ |
| **Q3** | 1.30 (1.02,1.68) | 1.33 (1.03,1.72) | 1.04 (0.85,1.27) | 1.28 (0.65,2.51) |
|  | *P* = **3.48×10^-2^** | *P* = **3.17×10^-2^** | *P* = 7.25×10^-1^ | *P* = 4.76×10^-1^ |
| **Q4** | 1.63 (1.28,2.07) | 1.75 (1.36,2.25) | 1.21 (0.99,1.47) | 1.02 (0.54,1.91) |
|  | *P* = **6.82×10^-5^** | *P* = **1.13×10^-5^** | *P* = 6.17×10^-2^ | *P* = 9.57×10^-1^ |
|  | *P_trend_* = **2.07×10^-5^** | *P_trend_* = **5.62×10^-6^** | *P_trend_* = **2.87×10^-3^** | *P_trend_* = 4.42×10^-1^ |

BMI = body mass index, CI = confidence interval, DMS = the Chinese National Diabetes and Metabolic Disorders Study, GRS = genotype risk score, OR = odds ratio, Q = quartile, T2D = type 2 diabetes.

Only T2D patients that did not receive the hypoglycemic treatment were included in the analysis. OR and 95% CI were reported for each T2D GRS quartile using logistic regression under an additive assumption using the following models: Model 1, age and sex were adjusted as co-variables; and Model 2, age, sex, and BMI were adjusted. *P* values were calculated for T2D GRS quartiles. *P_trend_* values were calculated for T2D GRS.

*P* values < 0.05 are denoted in bold and underlined.

Supplemental Table 4. Associations of type 2 diabetes genotype risk score with the obesity-related and glycemic quantitative traits in the hypoglycemic treatment naive type 2 diabetes patients of Chinese ancestry.

| **Trait** | **T2D GRS** | | **Q1** | **Q2** | **Q3** | **Q4** |
| --- | --- | --- | --- | --- | --- | --- |
|  | ***β* (SE)_Model 1_** | ***β* (SE)_Model 2_** |  |  |  |  |
| **Weight, kg** | -0.0031 (0.0011) | -0.0009 (0.0005) | 68.00 ( 60.00, 77.00) | 67.15 ( 60.75, 75.40) | 65.50 ( 59.00, 74.80) | 66.00 ( 58.80, 75.10) |
|  | *P* = **4.89×10^-3^** | *P* = 1.09×10^-1^ |  |  |  |  |
| **BMI, kg/m^2^** | -0.0022 (0.0010) | 0.0001 (0.0001) | 26.41 ( 23.75, 28.99) | 25.94 ( 23.62, 28.26) | 25.44 ( 23.36, 28.40) | 25.62 ( 23.53, 28.40) |
|  | *P* = **2.71×10^-2^** | *P* = 3.56×10^-1^ |  |  |  |  |
| **WC, cm** | -0.0018 (0.0008) | -0.0004 (0.0005) | 89.75 ( 82.00, 96.00) | 88.00 ( 82.00, 95.00) | 88.00 ( 81.00, 94.50) | 87.00 ( 80.00, 94.00) |
|  | *P* = **2.03×10^-2^** | *P* = 4.41×10^-1^ |  |  |  |  |
| **WHR** | -0.0011 (0.0005) | -0.0006 (0.0005) | 0.90 ( 0.85, 0.94) | 0.90 ( 0.85, 0.93) | 0.89 ( 0.85, 0.93) | 0.89 ( 0.85, 0.93) |
|  | *P* = **3.27×10^-2^** | *P* = 1.83×10^-1^ |  |  |  |  |
| **Fasting plasma glucose, mmol/l** | 0.0009 (0.0020) | 0.0012 (0.0020) | 7.12 ( 6.02, 8.30) | 7.13 ( 6.01, 8.44) | 7.12 ( 6.11, 8.30) | 7.16 ( 6.22, 8.40) |
|  | *P* = 6.35×10^-1^ | *P* = 5.47×10^-1^ |  |  |  |  |
| **30-min OGTT glucose, mmol/l** | 0.0039 (0.0020) | 0.0044 (0.0020) | 12.11 ( 10.31, 14.02) | 12.02 ( 10.32, 14.36) | 12.17 ( 10.44, 14.24) | 12.71 ( 10.47, 14.68) |
|  | *P* = **4.83×10^-2^** | *P* = **2.49×10^-2^** |  |  |  |  |
| **120-min OGTT glucose, mmol/l** | 0.0009 (0.0025) | 0.0015 (0.0025) | 12.60 ( 11.25, 15.38) | 13.01 ( 11.30, 16.08) | 12.88 ( 11.26, 16.02) | 12.72 ( 11.12, 15.89) |
|  | *P* = 7.15×10^-1^ | *P* = 5.48×10^-1^ |  |  |  |  |
| **Fasting serum insulin, mmol/l** | -0.0113 (0.0042) | -0.0082 (0.0040) | 9.09 ( 6.44, 12.63) | 8.72 ( 6.10, 12.35) | 8.58 ( 6.16, 11.67) | 8.68 ( 6.07, 12.18) |
|  | *P* = **7.43×10^-3^** | *P* = **4.33×10^-2^** |  |  |  |  |
| **30-min OGTT insulin, mmol/l** | -0.0181 (0.0061) | -0.0154 (0.0060) | 28.25 ( 15.57, 49.57) | 25.22 ( 13.57, 40.65) | 23.76 ( 14.19, 40.75) | 23.45 ( 14.45, 38.92) |
|  | *P* = **3.16×10^-3^** | *P* = **1.05×10^-4^** |  |  |  |  |
| **120-min OGTT insulin, mmol/l** | -0.0193 (0.0065) | -0.0148 (0.0063) | 43.31 ( 23.59, 79.72) | 36.32 ( 20.07, 67.98) | 39.56 ( 20.34, 66.31) | 36.35 ( 21.29, 60.85) |
|  | *P* = **3.02×10^-3^** | *P* = **1.83×10^-2^** |  |  |  |  |
| **HOMA-B, %** | -0.0151 (0.0057) | -0.0128 (0.0056) | 52.46 ( 33.70, 87.51) | 50.61 ( 31.42, 80.20) | 48.10 ( 30.86, 76.06) | 47.49 ( 30.15, 75.14) |
|  | *P* = **7.47×10^-3^** | *P* = **2.22×10^-2^** |  |  |  |  |
| **Insulinogenic index** | -0.0263 (0.0104) | -0.0244 (0.0104) | 3.88 ( 1.79, 8.19) | 3.22 ( 1.58, 6.33) | 3.24 ( 1.40, 6.90) | 3.01 ( 1.50, 6.44) |
|  | *P* = **1.15×10^-2^** | *P* = **1.91×10^-2^** |  |  |  |  |
| **HOMA-IR** | -0.0094 (0.0049) | -0.0060 (0.0047) | 2.96 ( 1.87, 4.35) | 2.88 ( 1.88, 4.43) | 2.82 ( 1.92, 4.20) | 2.93 ( 1.81, 4.42) |
|  | *P* = 5.61×10^-2^ | *P* = 2.05×10^-1^ |  |  |  |  |
| **ISIm** | 0.0110 (0.0044) | 0.0082 (0.0042) | 3.84 ( 2.57, 5.56) | 4.13 ( 2.78, 6.16) | 4.14 ( 2.91, 5.82) | 4.08 ( 2.84, 5.85) |
|  | *P* = **1.27×10^-2^** | *P* = **5.00×10^-2^** |  |  |  |  |

BMI = body mass index, DMS = the Chinese National Diabetes and Metabolic Disorders Study, GRS = genotype risk score, HOMA-B = the homeostasis model assessment for β-cell function, HOMA-IR = the homeostasis model assessment for insulin resistance, ISIm = Matsuda index, OGTT = oral glucose tolerance test, Q = quartile, SE = standard error; T2D = type 2 diabetes, WC = waist circumference, WHR = waist-hip-ratio.

All non-Gaussian distributed quantitative traits were natural logarithmically transformed to normalize distributions.

Only T2D patients that did not receive the hypoglycemic treatment were included in the analysis. *β* and SE were reported for T2D GRS with each trait using linear regression under an additive assumption using the following models: Model 1, age and sex were adjusted as co-variables; and Model 2, age, sex, and BMI were adjusted.

*P* values < 0.05 are shown in bold and underlined.

Quantitative measurements of the traits in each quartile are shown as median (interquartile range).

**Supplemental Table 5. Significant associations of type 2 diabetes-related genetic variants with the obesity-related and glycemic quantitative traits in type 2** diabetes patients of Chinese ancestry.

| **Trait** | **Gene** | **SNP** | **Chr** | **Position (Build 38)** | **Major/minor allele** | ***β* (SE)_Model 1_** | ***β* (SE)_Model 2_** |
| --- | --- | --- | --- | --- | --- | --- | --- |
|
| **Weight** | *PPARG* | rs1801282 | 3 | 12351626 | **C**/G | -0.0141 (0.0063) | -0.0019 (0.0034) |
|  |  |  |  |  |  | *P* = **2.61×10^-2^** | *P* = 5.70×10^-1^ |
| **Weight** | *CDKN2BAS* | rs10811661 | 9 | 22134095 | **T**/C | -0.0102 (0.0031) | -0.0022 (0.0017) |
|  |  |  |  |  |  | *P* = **1.01×10^-3^** | *P* = 1.92×10^-1^ |
| **Weight** | *CDC123/CAMK1D* | rs12779790 | 10 | 12286011 | A/**G** | -0.0084 (0.0039) | -0.0008 (0.0021) |
|  |  |  |  |  |  | *P* = **3.24×10^-2^** | *P* = 7.12×10^-1^ |
| **Weight** | *MTNRIB* | rs10830963 | 11 | 92975544 | C/**G** | -0.0070 (0.0031) | -0.0035 (0.0016) |
|  |  |  |  |  |  | *P* = **2.41×10^-2^** | *P* = **3.44×10^-2^** |
| **Weight** | *KCNQ1* | rs2237895 | 11 | 2835964 | A/**C** | -0.0099 (0.0034) | -0.0029 (0.0017) |
|  |  |  |  |  |  | *P* = **4.14×10^-3^** | *P* = 9.40×10^-2^ |
| **Weight** | *FTO* | rs8050136 | 16 | 53782363 | C/**A** | 0.0106 (0.0045) | 0.0022 (0.0024) |
|  |  |  |  |  |  | *P* = **1.86×10^-2^** | *P* = 3.54×10^-1^ |
| **Weight** | *FTO* | rs9939609 | 16 | 53786615 | T/**A** | 0.0093 (0.0045) | 0.0015 (0.0024) |
|  |  |  |  |  |  | *P* = **3.81×10^-2^** | *P* = 5.29×10^-1^ |
| **BMI** | *BCL11A* | rs243021 | 2 | 60357684 | **T**/C | -0.0012 (0.0030) | 0.0008 (0.0003) |
|  |  |  |  |  |  | *P* = 6.77×10^-1^ | *P* = **1.75×10^-2^** |
| **BMI** | *PPARG* | rs1801282 | 3 | 12351626 | **C**/G | -0.0132 (0.0057) | 0.0013 (0.0006) |
|  |  |  |  |  |  | *P* = **2.13×10^-2^** | *P* = **3.74×10^-2^** |
| **BMI** | *CDKAL1* | rs7756992 | 6 | 20679478 | **G**/A | -0.0056 (0.0028) | 0.0007 (0.0003) |
|  |  |  |  |  |  | *P* = **4.25×10^-2^** | *P* = **2.18×10^-2^** |
| **BMI** | *CDKN2BAS* | rs10811661 | 9 | 22134095 | **T**/C | -0.0091 (0.0028) | 0.0006 (0.0003) |
|  |  |  |  |  |  | *P* = **1.18×10^-3^** | *P* = 7.38×10^-2^ |
| **BMI** | *CDC123/CAMK1D* | rs12779790 | 10 | 12286011 | A/**G** | -0.0085 (0.0036) | -0.0001 (0.0004) |
|  |  |  |  |  |  | *P* = **1.70×10^-2^** | *P* = 7.65×10^-1^ |
| **BMI** | *KCNQ1* | rs2237895 | 11 | 2835964 | A/**C** | -0.0071 (0.0031) | -0.0001 (0.0003) |
|  |  |  |  |  |  | *P* = **2.28×10^-2^** | *P* = 8.52×10^-1^ |
| **BMI** | *FTO* | rs8050136 | 16 | 53782363 | C/**A** | 0.0100 (0.0041) | -0.0004 (0.0004) |
|  |  |  |  |  |  | *P* = **1.49×10^-2^** | *P* = 4.10×10^-1^ |
| **BMI** | *FTO* | rs9939609 | 16 | 53786615 | T/**A** | 0.0094 (0.0041) | -0.0003 (0.0004) |
|  |  |  |  |  |  | *P* = **2.15×10^-2^** | *P* = 4.76×10^-1^ |
| **WC** | *PPARG* | rs1801282 | 3 | 12351626 | **C**/G | -0.0097 (0.0045) | -0.0022 (0.0032) |
|  |  |  |  |  |  | *P* = **3.15×10^-2^** | *P* = 4.89×10^-1^ |
| **WC** | *WFS1* | rs10010131 | 4 | 6291188 | **G**/A | 0.0087 (0.0055) | 0.0081 (0.0039) |
|  |  |  |  |  |  | *P* = 1.12×10^-1^ | *P* = **3.68×10^-2^** |
| **WC** | *TP53INP1* | rs896854 | 8 | 94948283 | G/**A** | 0.0046 (0.0023) | 0.0047 (0.0016) |
|  |  |  |  |  |  | *P* = **4.70×10^-2^** | *P* = **4.15×10^-3^** |
| **WC** | *CDKN2BAS* | rs10811661 | 9 | 22134095 | **T**/C | -0.0043 (0.0022) | 0.0008 (0.0016) |
|  |  |  |  |  |  | *P* = **4.95×10^-2^** | *P* = 6.21×10^-1^ |
| **WC** | *CHCHD9* | rs13292136 | 9 | 79337213 | **C**/T | -0.0031 (0.0036) | -0.0052 (0.0026) |
|  |  |  |  |  |  | *P* = 3.98×10^-1^ | *P* = **4.28×10^-2^** |
| **WC** | *CDC123/CAMK1D* | rs12779790 | 10 | 12286011 | A/**G** | -0.0066 (0.0028) | -0.0016 (0.0020) |
|  |  |  |  |  |  | *P* = **1.89×10^-2^** | *P* = 4.22×10^-1^ |
| **WC** | *KCNQ1* | rs2237895 | 11 | 2835964 | A/**C** | -0.0068 (0.0024) | -0.0035 (0.0017) |
|  |  |  |  |  |  | *P* = **5.23×10^-3^** | *P* = **4.01×10^-2^** |
| **WC** | *FTO* | rs8050136 | 16 | 53782363 | C/**A** | 0.0085 (0.0032) | 0.0053 (0.0023) |
|  |  |  |  |  |  | *P* = **7.87×10^-3^** | *P* = **1.93×10^-2^** |
| **WC** | *FTO* | rs9939609 | 16 | 53786615 | T/**A** | 0.0081 (0.0032) | 0.0053 (0.0023) |
|  |  |  |  |  |  | *P* = **1.12×10^-2^** | *P* = **2.02×10^-2^** |
| **WHR** | *CDKN2BAS* | rs10811661 | 9 | 22134095 | **T**/C | 0.0014 (0.0014) | 0.0028 (0.0014) |
|  |  |  |  |  |  | *P* = 3.40×10^-1^ | *P* = **3.78×10^-2^** |
| **Fasting plasma glucose** | *GCKR* | rs780094 | 2 | 27518370 | A/**G** | 0.0129 (0.0059) | 0.0129 (0.0059) |
|  |  |  |  |  |  | *P* = **2.98×10^-2^** | *P* = **2.97×10^-2^** |
| **30-min OGTT glucose** | *KCNQ1* | rs2237895 | 11 | 2835964 | A/**C** | 0.0186 (0.0067) | 0.0193 (0.0067) |
|  |  |  |  |  |  | *P* = **5.52×10^-3^** | *P* = **3.81×10^-3^** |
| **30-min OGTT glucose** | *CENTD2* | rs1552224 | 11 | 72722053 | **T**/G | 0.0280 (0.0107) | 0.0287 (0.0107) |
|  |  |  |  |  |  | *P* = **8.88×10^-3^** | *P* = **7.33×10^-3^** |
| **120-min OGTT glucose** | *KCNQ1* | rs2237895 | 11 | 2835964 | A/**C** | 0.0199 (0.0083) | 0.0202 (0.0083) |
|  |  |  |  |  |  | *P* = **1.63×10^-2^** | *P* = **1.48×10^-2^** |
| **Fasting serum insulin** | *CENTD2* | rs1552224 | 11 | 72722053 | **T**/G | -0.0439 (0.0221) | -0.0351 (0.0213) |
|  |  |  |  |  |  | *P* = **4.67×10^-2^** | *P* = 9.92×10^-2^ |
| **30-min OGTT insulin** | *PPARG* | rs1801282 | 3 | 12351626 | **C**/G | -0.0906 (0.0365) | -0.0648 (0.0357) |
|  |  |  |  |  |  | *P* = **1.30×10^-2^** | *P* = 6.97×10^-2^ |
| **30-min OGTT insulin** | *WFS1* | rs10010131 | 4 | 6291188 | **G**/A | -0.1217 (0.0445) | -0.1231 (0.0434) |
|  |  |  |  |  |  | *P* = **6.26×10^-3^** | *P* = **4.59×10^-3^** |
| **30-min OGTT insulin** | *CDKAL1* | rs7756992 | 6 | 20679478 | **G**/A | -0.0453 (0.0177) | -0.0365 (0.0173) |
|  |  |  |  |  |  | *P* = **1.06×10^-2^** | *P* = **3.48×10^-2^** |
| **30-min OGTT insulin** | *CDKN2BAS* | rs10811661 | 9 | 22134095 | **T**/C | -0.0479 (0.0179) | -0.0367 (0.0175) |
|  |  |  |  |  |  | *P* = **7.33×10^-3^** | *P* = **3.57×10^-2^** |
| **30-min OGTT insulin** | *TCF2* | rs7501939 | 17 | 37741165 | C/**T** | -0.0419 (0.0195) | -0.0424 (0.0190) |
|  |  |  |  |  |  | *P* = **3.17×10^-2^** | *P* = **2.61×10^-2^** |
| **120-min OGTT insulin** | *WFS1* | rs10010131 | 4 | 6291188 | **G**/A | -0.1183 (0.0463) | -0.1227 (0.0449) |
|  |  |  |  |  |  | *P* = **1.06×10^-2^** | *P* = **6.27×10^-3^** |
| **120-min OGTT insulin** | *CDKN2BAS* | rs10811661 | 9 | 22134095 | **T**/C | -0.0638 (0.0186) | -0.0510 (0.0181) |
|  |  |  |  |  |  | *P* = **6.29×10^-4^** | *P* = **4.89×10^-3^** |
| **120-min OGTT insulin** | *ZFAND6* | rs11634397 | 15 | 80139880 | A/**G** | -0.0508 (0.0302) | -0.0607 (0.0293) |
|  |  |  |  |  |  | *P* = 9.26×10^-2^ | *P* = **3.82×10^-2^** |
| **HOMA-B** | *BCL11A* | rs243021 | 2 | 60357684 | **T**/C | -0.0477 (0.0183) | -0.0465 (0.0180) |
|  |  |  |  |  |  | *P* = **9.17×10^-3^** | *P* = **9.84×10^-3^** |
| **HOMA-B** | *HHEX* | rs1111875 | 10 | 92703125 | A/**G** | -0.0372 (0.0187) | -0.0337 (0.0184) |
|  |  |  |  |  |  | *P* = **4.68×10^-2^** | *P* = 6.73×10^-2^ |
| **HOMA-B** | *CENTD2* | rs1552224 | 11 | 72722053 | **T**/G | -0.0757 (0.0303) | -0.0676 (0.0298) |
|  |  |  |  |  |  | *P* = **1.24×10^-2^** | *P* = **2.34×10^-2^** |
| **Insulinogenic index** | *PPARG* | rs1801282 | 3 | 12351626 | **C**/G | -0.1278 (0.0645) | -0.0935 (0.0641) |
|  |  |  |  |  |  | *P* = **4.77×10^-2^** | *P* = 1.45×10^-1^ |
| **Insulinogenic index** | *WFS1* | rs10010131 | 4 | 6291188 | **G**/A | -0.1656 (0.0777) | -0.1752 (0.0770) |
|  |  |  |  |  |  | *P* = **3.30×10^-2^** | *P* = **2.30×10^-2^** |
| **Insulinogenic index** | *CDKN2BAS* | rs10811661 | 9 | 22134095 | **T**/C | -0.0694 (0.0315) | -0.0611 (0.0312) |
|  |  |  |  |  |  | *P* = **2.75×10^-2^** | *P* = 5.04×10^-2^ |
| **Insulinogenic index** | *KCNQ1* | rs2237895 | 11 | 2835964 | A/**C** | -0.0887 (0.0349) | -0.0820 (0.0347) |
|  |  |  |  |  |  | *P* = **1.11×10^-2^** | *P* = **1.81×10^-2^** |
| **Insulinogenic index** | *CENTD2* | rs1552224 | 11 | 72722053 | **T**/G | -0.1200 (0.0566) | -0.1156 (0.0561) |
|  |  |  |  |  |  | *P* = **3.40×10^-2^** | *P* = **3.96×10^-2^** |
| **ISIm** | *PPARG* | rs1801282 | 3 | 12351626 | **C**/G | 0.0561 (0.0267) | 0.0296 (0.0256) |
|  |  |  |  |  |  | *P* = **3.54×10^-2^** | *P* = 2.74×10^-1^ |

BMI = body mass index, DMS = the Chinese National Diabetes and Metabolic Disorders Study, HOMA-B = the homeostasis model assessment for β-cell function, HOMA-IR = the homeostasis model assessment for insulin resistance, ISIm = Matsuda index, OGTT = oral glucose tolerance test, SE = standard error, SNP = single nucleotide polymorphism, T2D = type 2 diabetes, WC = waist circumference, WHR = waist-hip-ratio.

All non-Gaussian distributed quantitative traits were natural logarithmically transformed to normalize distributions.

*β* and SE were reported for the T2D risk allele with the traits using linear regression under an additive assumption using the following models: Model 1, age and sex were adjusted as co-variables; and Model 2, age, sex, and BMI were adjusted.

Associations with *P* values < 0.05 are shown in bold and underlined.
